# Methyl 4-(4-chloro­phen­yl)-8-iodo-2-methyl-6-oxo-1,6-dihydro-4*H*-pyrimido[2,1-*b*]quinazoline-3-carboxyl­ate

**DOI:** 10.1107/S1600536812050787

**Published:** 2012-12-22

**Authors:** Susanta K. Nayak, K. N. Venugopala, Bharti Odhav

**Affiliations:** a Center for Nano Science and Technology@Polimi, Istituto Italiano di Tecnologia, Via Pascoli 70/3-20133 Milan, Italy; b Department of Biotechnology and Food Technology, Durban University of Technology, Durban 4001, South Africa

## Abstract

In the title compound, C_20_H_15_ClIN_3_O_3_, the dihedral angle between the quinazolinone ring system [r.m.s. deviation = 0.047 (2) Å] and the pendant benzene ring is 82.63 (11)°. The mol­ecular conformation is stabilized by intra­molecular C—H⋯O inter­actions. In the crystal, the mol­ecules are linked by N—H⋯O hydrogen bonds into chains along the *a-*axis direction. Another set of chains propagating along [101] is formed due to inter­molecular I⋯Cl short contacts of 3.427 (1) Å, thus giving layers parallel to (010). The layers are connected by C—H⋯π and π–π inter­actions, the shortest distance between the centroids of aromatic rings being 3.8143 (16) Å.

## Related literature

For crystal structures of dihydro­pyrimidines, see: Nayak *et al.* (2010 ▶, 2011*a* ▶,*b* ▶,*c* ▶); Venugopala *et al.* (2012 ▶). For applications of dihydro­pyrimidines, see: Kappe (2000 ▶). For halogen-involving inter­actions, see: Nayak *et al.* (2011*b* ▶).
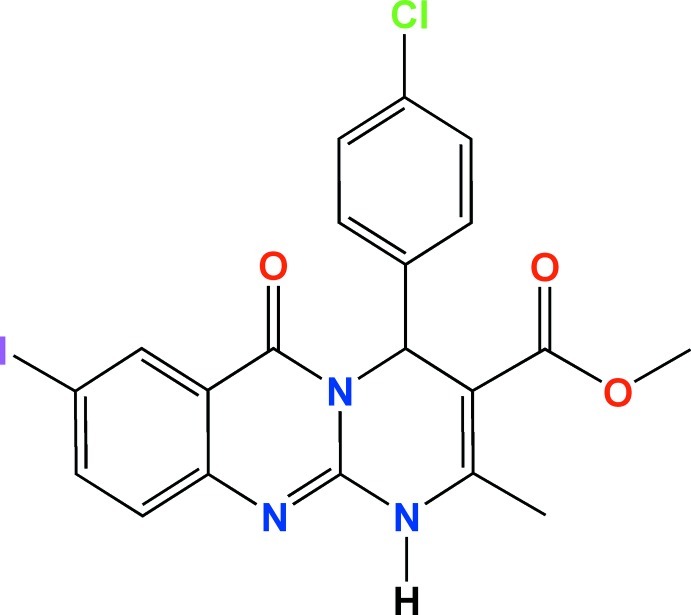


## Experimental

### 

#### Crystal data

C_20_H_15_ClIN_3_O_3_
*M* *_r_* = 507.70
Triclinic, 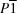

*a* = 7.3443 (15) Å
*b* = 10.847 (2) Å
*c* = 12.475 (3) Å
α = 106.66 (3)°
β = 103.53 (2)°
γ = 92.79 (3)°
*V* = 918.5 (4) Å^3^
*Z* = 2
Mo *K*α radiation
μ = 1.92 mm^−1^
*T* = 173 K
0.25 × 0.14 × 0.12 mm

#### Data collection

Bruker APEXII Kappa DUO CCD diffractometer
Absorption correction: multi-scan (*SADABS*; Bruker, 2008 ▶) *T*_min_ = 0.646, *T*_max_ = 0.803
7109 measured reflections
3602 independent reflections
3147 reflections with *I* > 2σ(*I*)
*R*_int_ = 0.017

#### Refinement

*R*[*F* ^2^ > 2σ(*F* ^2^)] = 0.024
*wR*(*F* ^2^) = 0.059
*S* = 1.09
3602 reflections
255 parameters
H-atom parameters constrained
Δρ_max_ = 0.89 e Å^−3^
Δρ_min_ = −0.52 e Å^−3^

### 

Data collection: *APEX2* (Bruker, 2008 ▶); cell refinement: *SAINT* (Bruker, 2008 ▶); data reduction: *SAINT*; program(s) used to solve structure: *SHELXS97* (Sheldrick, 2008 ▶); program(s) used to refine structure: *SHELXL97* (Sheldrick, 2008 ▶); molecular graphics: *ORTEP-3 for Windows* (Farrugia, 2012 ▶) and *Mercury* (Macrae *et al.*, 2008 ▶); software used to prepare material for publication: *PLATON* (Spek, 2009 ▶) and *PARST* (Nardelli, 1995 ▶).

## Supplementary Material

Crystal structure: contains datablock(s) global, I. DOI: 10.1107/S1600536812050787/yk2082sup1.cif

Structure factors: contains datablock(s) I. DOI: 10.1107/S1600536812050787/yk2082Isup2.hkl

Supplementary material file. DOI: 10.1107/S1600536812050787/yk2082Isup3.cml

Additional supplementary materials:  crystallographic information; 3D view; checkCIF report

## Figures and Tables

**Table 1:** Hydrogen-bond geometry (Å, °) *Cg*1 is the centroid of the C7–C12 ring.

*D*—H⋯*A*	*D*—H	H⋯*A*	*D*⋯*A*	*D*—H⋯*A*
N1—H1⋯O3^i^	0.88	2.04	2.903 (3)	167
C5—H5*A*⋯O1	0.98	2.22	2.807 (4)	117
C8—H8⋯O2	0.95	2.49	3.167 (4)	128
C1—H1*B*⋯*Cg*1^ii^	0.98	2.67	3.647 (4)	175

Symmetry codes: (i) 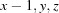; (ii) 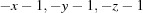.

**Fig. 1.**
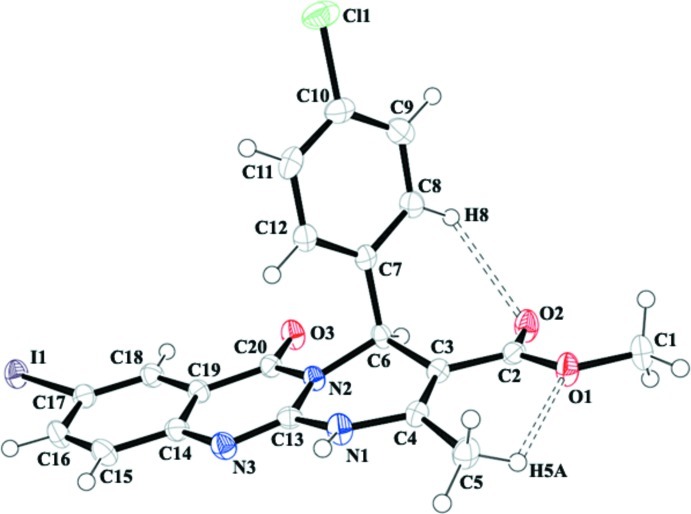
A view of the title compound with the atom numbering scheme and displacement ellipsoids for non-H atoms drawn at the 50% probability level. The intramolecular C—H···O interactions are shown by dashed lines.

**Fig. 2.**
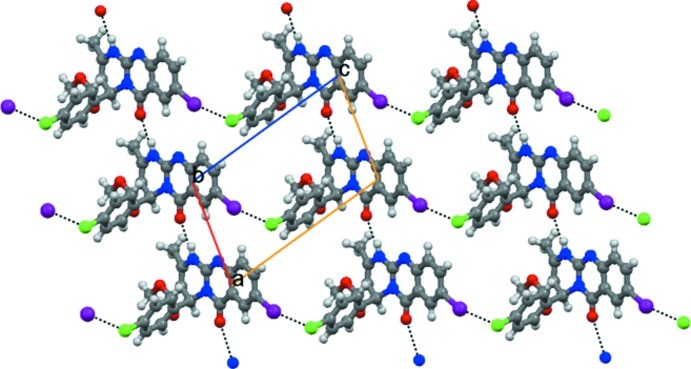
Intermolecular N—H···O hydrogen bonds and short contacts I···Cl forming layers parallel to (010).

## supplementary crystallographic information

### Comment

In continuation of our work on the pharmacological properties and single-crystal X-ray studies (Nayak *et al.*, 2010, 2011*a*,*b*,*c*; Venugopala *et al.*, 2012) on dihydropyrimidine derivatives, we synthesized the title compound as a potential anti-malarial agent. Here we are reporting the single-crystal structure of the title compound.

The conformation of the title molecule is stabilized by intramolecular C—H···O interactions, and the dihedral angle between the planes of the 4-chlorophenyl and iodophenyl groups is 80.3 (2)° (Fig. 1). The crystal structure is stabilized by N—H···O infinite hydrogen bond chains parallel to [0 1 0]. Halogen-involving short contacts I···Cl [3.427 (2) Å, θ_1_= 166.1 (2)°; θ_2_= 90.5 (2)°, symmetry code: *x* + 1, *y*, *z* + 1, Type II (Nayak *et al.*, 2011*b*)] form infinite chains orthogonal to hydrogen bond chains which lead to two-dimensional molecular assembly (Fig. 2). Further, the C—H···π [2.67 Å,*Cg1* = Centroid of six membered ring C7—C12; Table 1] and π–π [*Cg2*···*Cg2* = 3.814 (2) Å, symmetry code: –*X*,1-Y,-*Z*; *Cg2* = Centroid of six membered ring N2/C13/N3/C14/C19/C20] interactions enhance the stability of three-dimensional molecular assembly.

### Experimental

A mixture of methyl-2-chloro-4-(4-chlorophenyl)-6-methyl-1,4- dihydropyrimidine-5-carboxylate (1 mmol), 2-amino-5-iodobenzoic acid (1 mmol) and methanamine (1 mmol) in 2-propanol (5 ml) was refluxed for 12 h. The reaction completion was monitored by TLC. The reaction medium was cooled to room temperature, the product was filtered, washed with cold 2-propanol and dried to obtain the crude product. The product was purified by recrystallization using ethanol in 66% yield as a brown solid (m. p. 467 (2) K). Crystals suitable for single-crystal X-ray study were obtained from methanol and tetrahydrofuran (1:1) solvent using slow evaporation at room temperature.

### Refinement

All H atoms were positioned geometrically with N—H = 0.88 Å, C—H = 0.95–1.00 Å and refined using a riding model with *U*_iso_(H) = 1.2 *U*_eq_(C/N) except for the methyl group where *U*_iso_(H) = 1.5 *U*_eq_(C).

### Crystal data

**Table:**

C_20_H_15_ClIN_3_O_3_	*Z* = 2
*M**_r_* = 507.70	*F*(000) = 500
Triclinic, *P*1	*D*_x_ = 1.836 Mg m^−^^3^
Hall symbol: -P 1	Melting point: 467(2) K
*a* = 7.3443 (15) Å	Mo *K*α radiation, λ = 0.71073 Å
*b* = 10.847 (2) Å	Cell parameters from 560 reflections
*c* = 12.475 (3) Å	θ = 2.9–26.0°
α = 106.66 (3)°	µ = 1.92 mm^−^^1^
β = 103.53 (2)°	*T* = 173 K
γ = 92.79 (3)°	Plate, yellow
*V* = 918.5 (4) Å^3^	0.25 × 0.14 × 0.12 mm

### Data collection

**Table:**

Bruker APEXII Kappa DUO CCD diffractometer	3602 independent reflections
Radiation source: fine-focus sealed tube	3147 reflections with *I* > 2σ(*I*)
Graphite monochromator	*R*_int_ = 0.017
0.5° φ scans and ω scans	θ_max_ = 26.0°, θ_min_ = 2.9°
Absorption correction: multi-scan (*SADABS*; Bruker, 2008)	*h* = −9→9
*T*_min_ = 0.646, *T*_max_ = 0.803	*k* = −13→13
7109 measured reflections	*l* = −15→15

### Refinement

**Table:**

Refinement on *F*^2^	Primary atom site location: structure-invariant direct methods
Least-squares matrix: full	Secondary atom site location: difference Fourier map
*R*[*F*^2^ > 2σ(*F*^2^)] = 0.024	Hydrogen site location: inferred from neighbouring sites
*wR*(*F*^2^) = 0.059	H-atom parameters constrained
*S* = 1.09	*w* = 1/[σ^2^(*F*_o_^2^) + (0.0285*P*)^2^ + 0.4298*P*] where *P* = (*F*_o_^2^ + 2*F*_c_^2^)/3
3602 reflections	(Δ/σ)_max_ = 0.003
255 parameters	Δρ_max_ = 0.89 e Å^−^^3^
0 restraints	Δρ_min_ = −0.52 e Å^−^^3^

### Special details

**Table:**

Geometry. All s.u.'s (except the s.u. in the dihedral angle between two l.s. planes) are estimated using the full covariance matrix. The cell s.u.'s are taken into account individually in the estimation of s.u.'s in distances, angles and torsion angles; correlations between s.u.'s in cell parameters are only used when they are defined by crystal symmetry. An approximate (isotropic) treatment of cell s.u.'s is used for estimating s.u.'s involving l.s. planes.
Refinement. Refinement of *F*^2^ against ALL reflections. The weighted *R*-factor *wR* and goodness of fit *S* are based on *F*^2^, conventional *R*-factors *R* are based on *F*, with *F* set to zero for negative *F*^2^. The threshold expression of *F*^2^ > 2σ(*F*^2^) is used only for calculating *R*-factors(gt) *etc*. and is not relevant to the choice of reflections for refinement. *R*-factors based on *F*^2^ are statistically about twice as large as those based on *F*, and *R*- factors based on ALL data will be even larger.

### Fractional atomic coordinates and isotropic or equivalent isotropic displacement parameters (Å^2^)

**Table:**

	*x*	*y*	*z*	*U*_iso_*/*U*_eq_	
I1	1.42667 (2)	0.047532 (17)	1.168961 (15)	0.02807 (8)	
Cl1	0.75652 (11)	−0.07966 (7)	0.33638 (6)	0.03791 (19)	
O1	0.6050 (3)	0.62853 (18)	0.61509 (16)	0.0272 (4)	
O2	0.9047 (3)	0.59136 (19)	0.62752 (17)	0.0287 (4)	
O3	1.2104 (2)	0.34709 (18)	0.86038 (16)	0.0250 (4)	
N1	0.5905 (3)	0.4115 (2)	0.84598 (19)	0.0238 (5)	
H1	0.4842	0.3915	0.8617	0.029*	
N2	0.9040 (3)	0.3772 (2)	0.85309 (17)	0.0184 (4)	
N3	0.7400 (3)	0.3144 (2)	0.97648 (19)	0.0235 (5)	
C1	0.6278 (4)	0.7087 (3)	0.5443 (3)	0.0312 (7)	
H1A	0.7308	0.7789	0.5874	0.047*	
H1B	0.5104	0.7457	0.5232	0.047*	
H1C	0.6577	0.6562	0.4738	0.047*	
C2	0.7579 (4)	0.5753 (2)	0.6529 (2)	0.0205 (6)	
C3	0.7317 (3)	0.4945 (2)	0.7260 (2)	0.0185 (5)	
C4	0.5854 (4)	0.4883 (2)	0.7733 (2)	0.0208 (5)	
C5	0.4109 (4)	0.5552 (3)	0.7604 (3)	0.0270 (6)	
H5A	0.4379	0.6358	0.7432	0.041*	
H5B	0.3712	0.5751	0.8326	0.041*	
H5C	0.3097	0.4983	0.6969	0.041*	
C6	0.8863 (3)	0.4093 (2)	0.7435 (2)	0.0182 (5)	
H6	1.0084	0.4588	0.7491	0.022*	
C7	0.8535 (3)	0.2847 (2)	0.6416 (2)	0.0180 (5)	
C8	0.9062 (4)	0.2870 (3)	0.5422 (2)	0.0230 (6)	
H8	0.9657	0.3653	0.5392	0.028*	
C9	0.8728 (4)	0.1763 (3)	0.4471 (2)	0.0268 (6)	
H9	0.9064	0.1790	0.3787	0.032*	
C10	0.7903 (4)	0.0623 (3)	0.4531 (2)	0.0261 (6)	
C11	0.7360 (4)	0.0575 (3)	0.5507 (2)	0.0270 (6)	
H11	0.6781	−0.0213	0.5536	0.032*	
C12	0.7672 (4)	0.1693 (3)	0.6445 (2)	0.0231 (6)	
H12	0.7290	0.1669	0.7116	0.028*	
C13	0.7476 (4)	0.3651 (2)	0.8947 (2)	0.0192 (5)	
C14	0.8986 (4)	0.2618 (2)	1.0197 (2)	0.0212 (6)	
C15	0.8893 (4)	0.1949 (3)	1.0998 (2)	0.0262 (6)	
H15	0.7788	0.1913	1.1265	0.031*	
C16	1.0389 (4)	0.1347 (3)	1.1397 (2)	0.0264 (6)	
H16	1.0300	0.0875	1.1921	0.032*	
C17	1.2046 (4)	0.1424 (3)	1.1035 (2)	0.0233 (6)	
C18	1.2198 (4)	0.2091 (3)	1.0265 (2)	0.0227 (6)	
H18	1.3325	0.2143	1.0022	0.027*	
C19	1.0655 (4)	0.2691 (2)	0.9845 (2)	0.0194 (5)	
C20	1.0732 (4)	0.3341 (2)	0.8977 (2)	0.0194 (5)	

### Atomic displacement parameters (Å^2^)

**Table:**

	*U*^11^	*U*^22^	*U*^33^	*U*^12^	*U*^13^	*U*^23^
I1	0.02738 (11)	0.03098 (12)	0.02945 (11)	0.00738 (8)	0.00520 (8)	0.01593 (8)
Cl1	0.0363 (4)	0.0311 (4)	0.0339 (4)	0.0142 (3)	−0.0019 (3)	−0.0018 (3)
O1	0.0242 (10)	0.0311 (11)	0.0342 (11)	0.0093 (8)	0.0084 (9)	0.0202 (9)
O2	0.0265 (11)	0.0315 (11)	0.0400 (12)	0.0078 (8)	0.0166 (9)	0.0220 (9)
O3	0.0167 (9)	0.0348 (11)	0.0314 (10)	0.0061 (8)	0.0103 (8)	0.0185 (9)
N1	0.0154 (11)	0.0320 (13)	0.0320 (13)	0.0073 (9)	0.0111 (10)	0.0173 (10)
N2	0.0153 (11)	0.0229 (12)	0.0202 (11)	0.0031 (9)	0.0065 (9)	0.0097 (9)
N3	0.0213 (12)	0.0289 (13)	0.0247 (12)	0.0064 (10)	0.0095 (10)	0.0115 (10)
C1	0.0335 (16)	0.0341 (17)	0.0321 (16)	0.0071 (13)	0.0055 (13)	0.0215 (13)
C2	0.0221 (14)	0.0174 (13)	0.0205 (13)	0.0040 (11)	0.0037 (11)	0.0044 (11)
C3	0.0159 (13)	0.0186 (13)	0.0205 (13)	0.0023 (10)	0.0037 (10)	0.0060 (11)
C4	0.0209 (14)	0.0191 (13)	0.0231 (13)	0.0042 (11)	0.0047 (11)	0.0080 (11)
C5	0.0230 (14)	0.0303 (15)	0.0363 (16)	0.0118 (12)	0.0140 (13)	0.0168 (13)
C6	0.0167 (12)	0.0228 (14)	0.0194 (13)	0.0034 (10)	0.0069 (10)	0.0113 (11)
C7	0.0121 (12)	0.0223 (14)	0.0220 (13)	0.0069 (10)	0.0039 (10)	0.0103 (11)
C8	0.0208 (14)	0.0261 (15)	0.0264 (14)	0.0047 (11)	0.0102 (11)	0.0113 (12)
C9	0.0237 (14)	0.0353 (17)	0.0237 (14)	0.0103 (13)	0.0078 (12)	0.0100 (12)
C10	0.0210 (14)	0.0263 (15)	0.0251 (14)	0.0107 (12)	−0.0004 (12)	0.0027 (12)
C11	0.0242 (15)	0.0211 (15)	0.0360 (16)	0.0041 (12)	0.0032 (12)	0.0128 (13)
C12	0.0227 (14)	0.0247 (15)	0.0254 (14)	0.0046 (11)	0.0077 (12)	0.0113 (12)
C13	0.0176 (13)	0.0205 (13)	0.0199 (13)	0.0040 (10)	0.0070 (11)	0.0046 (11)
C14	0.0221 (14)	0.0218 (14)	0.0198 (13)	0.0045 (11)	0.0056 (11)	0.0058 (11)
C15	0.0260 (15)	0.0340 (16)	0.0237 (14)	0.0041 (12)	0.0122 (12)	0.0119 (12)
C16	0.0308 (15)	0.0312 (16)	0.0217 (14)	0.0047 (12)	0.0079 (12)	0.0141 (12)
C17	0.0256 (14)	0.0240 (14)	0.0202 (13)	0.0057 (11)	0.0042 (11)	0.0075 (11)
C18	0.0223 (14)	0.0247 (14)	0.0220 (14)	0.0044 (11)	0.0066 (11)	0.0079 (11)
C19	0.0183 (13)	0.0212 (13)	0.0192 (13)	0.0014 (11)	0.0052 (10)	0.0070 (11)
C20	0.0187 (13)	0.0193 (13)	0.0190 (13)	0.0023 (10)	0.0029 (11)	0.0058 (11)

### Geometric parameters (Å, º)

**Table:**

I1—C17	2.099 (3)	C5—H5C	0.9800
Cl1—C10	1.750 (3)	C6—C7	1.530 (4)
O1—C2	1.338 (3)	C6—H6	1.0000
O1—C1	1.436 (3)	C7—C12	1.389 (4)
O2—C2	1.211 (3)	C7—C8	1.390 (3)
O3—C20	1.223 (3)	C8—C9	1.389 (4)
N1—C13	1.363 (3)	C8—H8	0.9500
N1—C4	1.392 (3)	C9—C10	1.379 (4)
N1—H1	0.8800	C9—H9	0.9500
N2—C13	1.382 (3)	C10—C11	1.380 (4)
N2—C20	1.398 (3)	C11—C12	1.388 (4)
N2—C6	1.483 (3)	C11—H11	0.9500
N3—C13	1.301 (3)	C12—H12	0.9500
N3—C14	1.386 (3)	C14—C19	1.401 (4)
C1—H1A	0.9800	C14—C15	1.404 (4)
C1—H1B	0.9800	C15—C16	1.372 (4)
C1—H1C	0.9800	C15—H15	0.9500
C2—C3	1.469 (3)	C16—C17	1.401 (4)
C3—C4	1.349 (3)	C16—H16	0.9500
C3—C6	1.515 (3)	C17—C18	1.379 (4)
C4—C5	1.502 (4)	C18—C19	1.405 (4)
C5—H5A	0.9800	C18—H18	0.9500
C5—H5B	0.9800	C19—C20	1.461 (3)
			
C2—O1—C1	115.4 (2)	C9—C8—H8	119.6
C13—N1—C4	125.0 (2)	C7—C8—H8	119.6
C13—N1—H1	117.5	C10—C9—C8	119.3 (3)
C4—N1—H1	117.5	C10—C9—H9	120.4
C13—N2—C20	121.4 (2)	C8—C9—H9	120.4
C13—N2—C6	120.6 (2)	C9—C10—C11	121.1 (3)
C20—N2—C6	116.72 (19)	C9—C10—Cl1	119.8 (2)
C13—N3—C14	116.7 (2)	C11—C10—Cl1	119.1 (2)
O1—C1—H1A	109.5	C10—C11—C12	119.1 (3)
O1—C1—H1B	109.5	C10—C11—H11	120.4
H1A—C1—H1B	109.5	C12—C11—H11	120.4
O1—C1—H1C	109.5	C11—C12—C7	120.9 (2)
H1A—C1—H1C	109.5	C11—C12—H12	119.5
H1B—C1—H1C	109.5	C7—C12—H12	119.5
O2—C2—O1	122.5 (2)	N3—C13—N1	118.2 (2)
O2—C2—C3	123.1 (2)	N3—C13—N2	125.2 (2)
O1—C2—C3	114.4 (2)	N1—C13—N2	116.6 (2)
C4—C3—C2	126.5 (2)	N3—C14—C19	122.9 (2)
C4—C3—C6	119.8 (2)	N3—C14—C15	118.4 (2)
C2—C3—C6	113.7 (2)	C19—C14—C15	118.7 (2)
C3—C4—N1	118.3 (2)	C16—C15—C14	120.4 (2)
C3—C4—C5	129.1 (2)	C16—C15—H15	119.8
N1—C4—C5	112.6 (2)	C14—C15—H15	119.8
C4—C5—H5A	109.5	C15—C16—C17	120.3 (2)
C4—C5—H5B	109.5	C15—C16—H16	119.8
H5A—C5—H5B	109.5	C17—C16—H16	119.8
C4—C5—H5C	109.5	C18—C17—C16	120.8 (2)
H5A—C5—H5C	109.5	C18—C17—I1	121.37 (19)
H5B—C5—H5C	109.5	C16—C17—I1	117.80 (19)
N2—C6—C3	111.06 (19)	C17—C18—C19	118.7 (2)
N2—C6—C7	110.0 (2)	C17—C18—H18	120.6
C3—C6—C7	111.7 (2)	C19—C18—H18	120.6
N2—C6—H6	108.0	C14—C19—C18	121.0 (2)
C3—C6—H6	108.0	C14—C19—C20	118.9 (2)
C7—C6—H6	108.0	C18—C19—C20	120.0 (2)
C12—C7—C8	118.8 (2)	O3—C20—N2	120.3 (2)
C12—C7—C6	121.6 (2)	O3—C20—C19	125.0 (2)
C8—C7—C6	119.6 (2)	N2—C20—C19	114.7 (2)
C9—C8—C7	120.7 (3)		
			
C1—O1—C2—O2	−1.3 (4)	C6—C7—C12—C11	179.1 (2)
C1—O1—C2—C3	179.5 (2)	C14—N3—C13—N1	176.6 (2)
O2—C2—C3—C4	168.5 (3)	C14—N3—C13—N2	−4.1 (4)
O1—C2—C3—C4	−12.4 (4)	C4—N1—C13—N3	168.0 (2)
O2—C2—C3—C6	−13.4 (4)	C4—N1—C13—N2	−11.4 (4)
O1—C2—C3—C6	165.8 (2)	C20—N2—C13—N3	0.0 (4)
C2—C3—C4—N1	−176.2 (2)	C6—N2—C13—N3	166.5 (2)
C6—C3—C4—N1	5.7 (4)	C20—N2—C13—N1	179.3 (2)
C2—C3—C4—C5	3.0 (5)	C6—N2—C13—N1	−14.2 (3)
C6—C3—C4—C5	−175.0 (3)	C13—N3—C14—C19	4.3 (4)
C13—N1—C4—C3	15.7 (4)	C13—N3—C14—C15	−173.8 (2)
C13—N1—C4—C5	−163.7 (2)	N3—C14—C15—C16	176.1 (3)
C13—N2—C6—C3	31.8 (3)	C19—C14—C15—C16	−2.0 (4)
C20—N2—C6—C3	−161.1 (2)	C14—C15—C16—C17	1.8 (4)
C13—N2—C6—C7	−92.5 (3)	C15—C16—C17—C18	−0.7 (4)
C20—N2—C6—C7	74.6 (3)	C15—C16—C17—I1	179.9 (2)
C4—C3—C6—N2	−27.2 (3)	C16—C17—C18—C19	−0.1 (4)
C2—C3—C6—N2	154.5 (2)	I1—C17—C18—C19	179.16 (19)
C4—C3—C6—C7	96.0 (3)	N3—C14—C19—C18	−176.9 (2)
C2—C3—C6—C7	−82.2 (3)	C15—C14—C19—C18	1.1 (4)
N2—C6—C7—C12	27.4 (3)	N3—C14—C19—C20	−0.5 (4)
C3—C6—C7—C12	−96.5 (3)	C15—C14—C19—C20	177.5 (2)
N2—C6—C7—C8	−154.5 (2)	C17—C18—C19—C14	−0.1 (4)
C3—C6—C7—C8	81.7 (3)	C17—C18—C19—C20	−176.4 (2)
C12—C7—C8—C9	0.2 (4)	C13—N2—C20—O3	−179.2 (2)
C6—C7—C8—C9	−178.0 (2)	C6—N2—C20—O3	13.8 (3)
C7—C8—C9—C10	−1.5 (4)	C13—N2—C20—C19	3.8 (3)
C8—C9—C10—C11	1.7 (4)	C6—N2—C20—C19	−163.2 (2)
C8—C9—C10—Cl1	−177.3 (2)	C14—C19—C20—O3	179.8 (3)
C9—C10—C11—C12	−0.6 (4)	C18—C19—C20—O3	−3.8 (4)
Cl1—C10—C11—C12	178.3 (2)	C14—C19—C20—N2	−3.4 (3)
C10—C11—C12—C7	−0.7 (4)	C18—C19—C20—N2	173.0 (2)
C8—C7—C12—C11	0.9 (4)		

### Hydrogen-bond geometry (Å, º)

Cg1 is the centroid of the C7–C12 ring.

**Table:**

*D*—H···*A*	*D*—H	H···*A*	*D*···*A*	*D*—H···*A*
N1—H1···O3^i^	0.88	2.04	2.903 (3)	167
C5—H5*A*···O1	0.98	2.22	2.807 (4)	117
C8—H8···O2	0.95	2.49	3.167 (4)	128
C1—H1*B*···*Cg*1^ii^	0.98	2.67	3.647 (4)	175

Symmetry codes: (i) *x*−1, *y*, *z*; (ii) −*x*−1, −*y*−1, −*z*−1.
